# Supplementary figures and images for: Novel secreted STPKLRR from Vibrio splendidus AJ01 promotes pathogen internalization via mediating tropomodulin phosphorylation dependent cytoskeleton rearrangement
Source: PLoS Pathog. 2023 May 22;19(5):e1011419. doi: 10.1371/journal.ppat.1011419 (PMC10237653; doi:10.1371/journal.ppat.1011419)

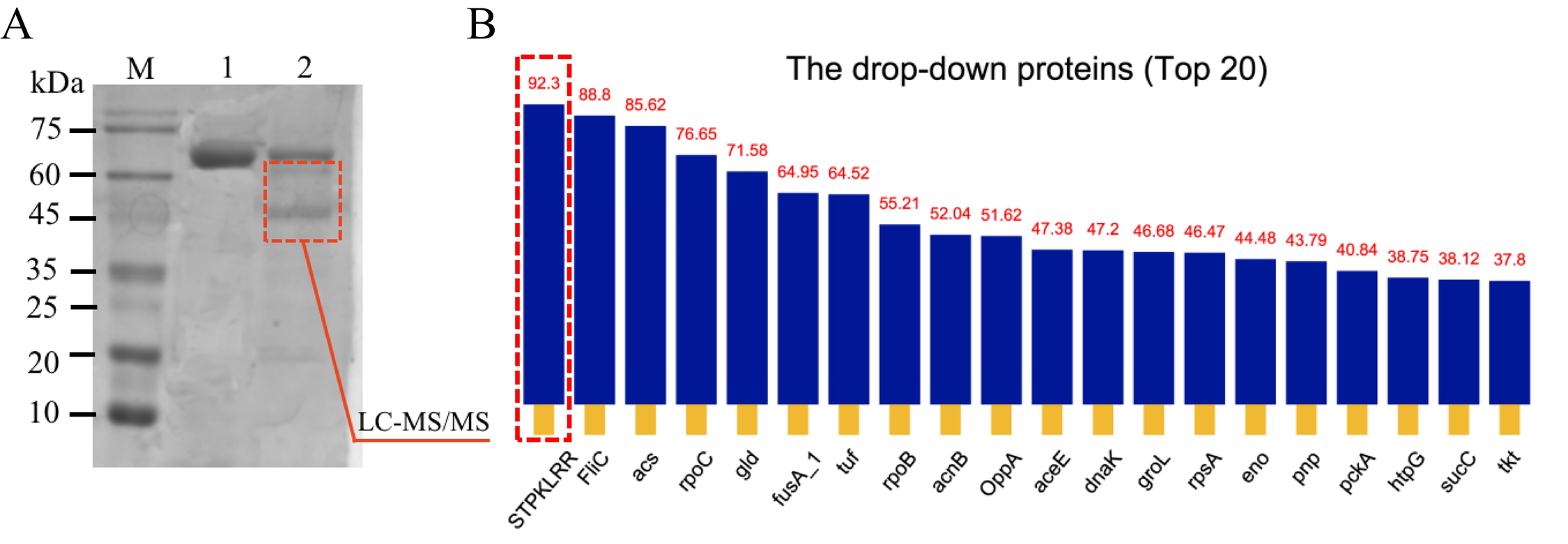

Supplement: S1 Fig — (A) Differential bands were detected by using SDS-PAGE. (B) The differential bands were further characterized through mass spectrometry, and the top 20 AjTmod-interacting proteins are listed. (TIF) [file ppat.1011419.s001.tif]

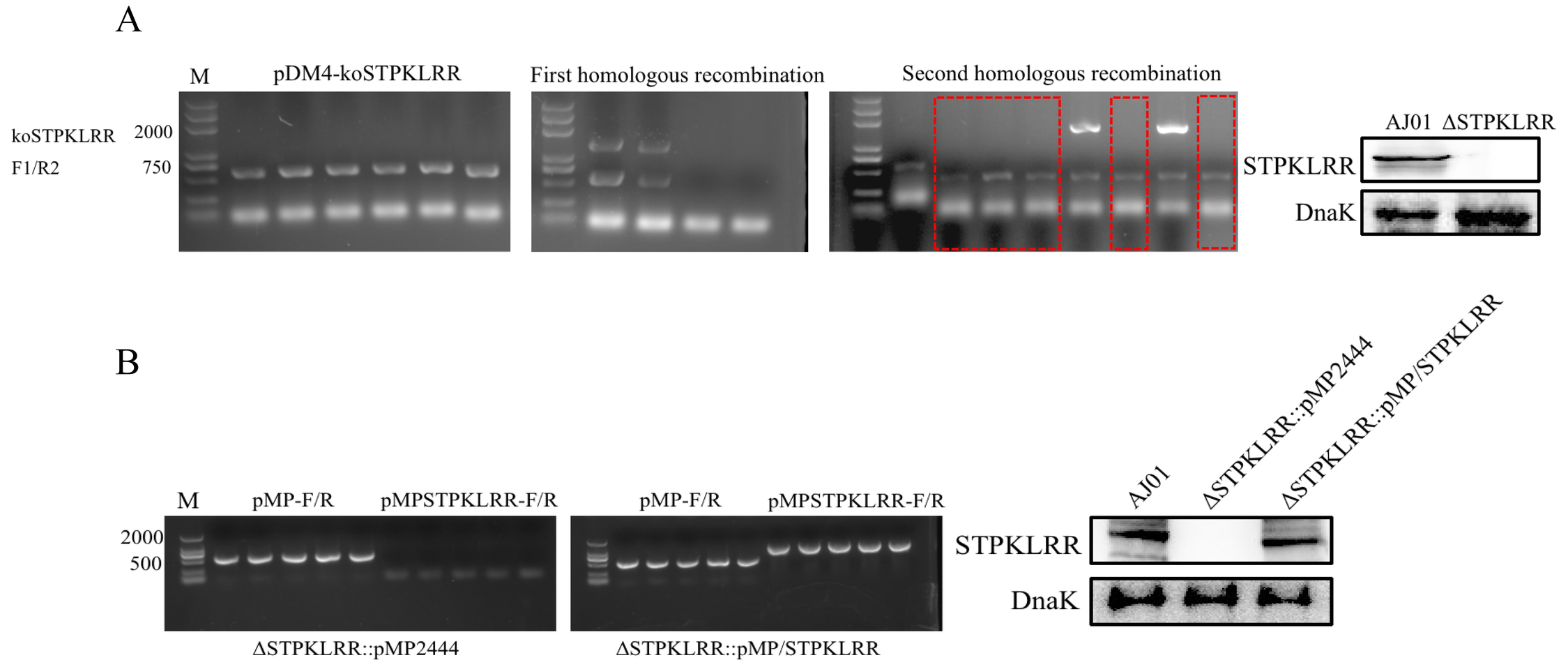

Supplement: S2 Fig — (A) Construction of the STPKLRR mutant strain ΔSTPKLRR. Left panel: The STPKLRR mutant was successfully constructed by two homologous recombination through bacterial conjugation. Right panel: Validation of STPKLRR knockdown by western blotting. (B) Construction of the complemented strain ΔSTPKLRR::pMP-STPKLRR, and the control complemented strain ΔSTPKLRR::pMP2444. Left panel: The plasmid pMP/STPKLRR and the empty plasmid pMP2444 were successfully transferred into the STPKLRR mutant strain ΔSTPKLRR through bacterial conjugation. Right panel: Validation of STPKLRR complement by western blotting. (TIF) [file ppat.1011419.s002.tif]

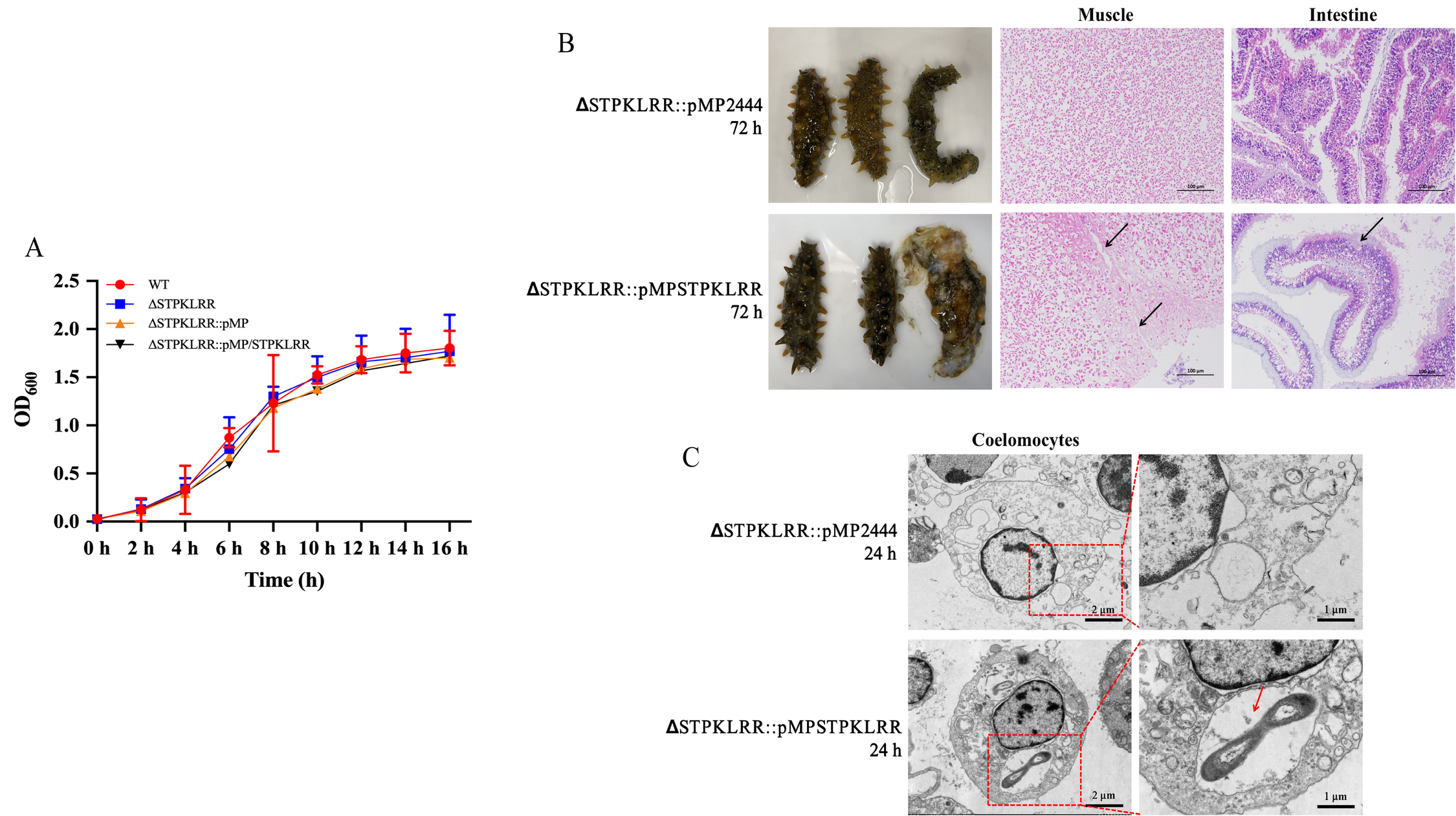

Supplement: S3 Fig — (A) No significant growth difference was detected among AJ01, ΔSTPKLRR, ΔSTPKLRR::pMP2444, and ΔSTPKLRR::pMP/STPKLRR. (B) Muscles, intestines were subjected to histological observation at 72 h after ΔSTPKLRR::pMP2444 and ΔSTPKLRR::pMPSTPKLRR infection to further confirm the pathogenic effect. Black arrows represent areas of tissue damage. Scale bar, 100 μm. (C) Intracellular ΔSTPKLRR::pMP2444 and ΔSTPKLRR::pMPSTPKLRR (red arrows) was detected by transmission electron microscopy. (TIF) [file ppat.1011419.s003.tif]

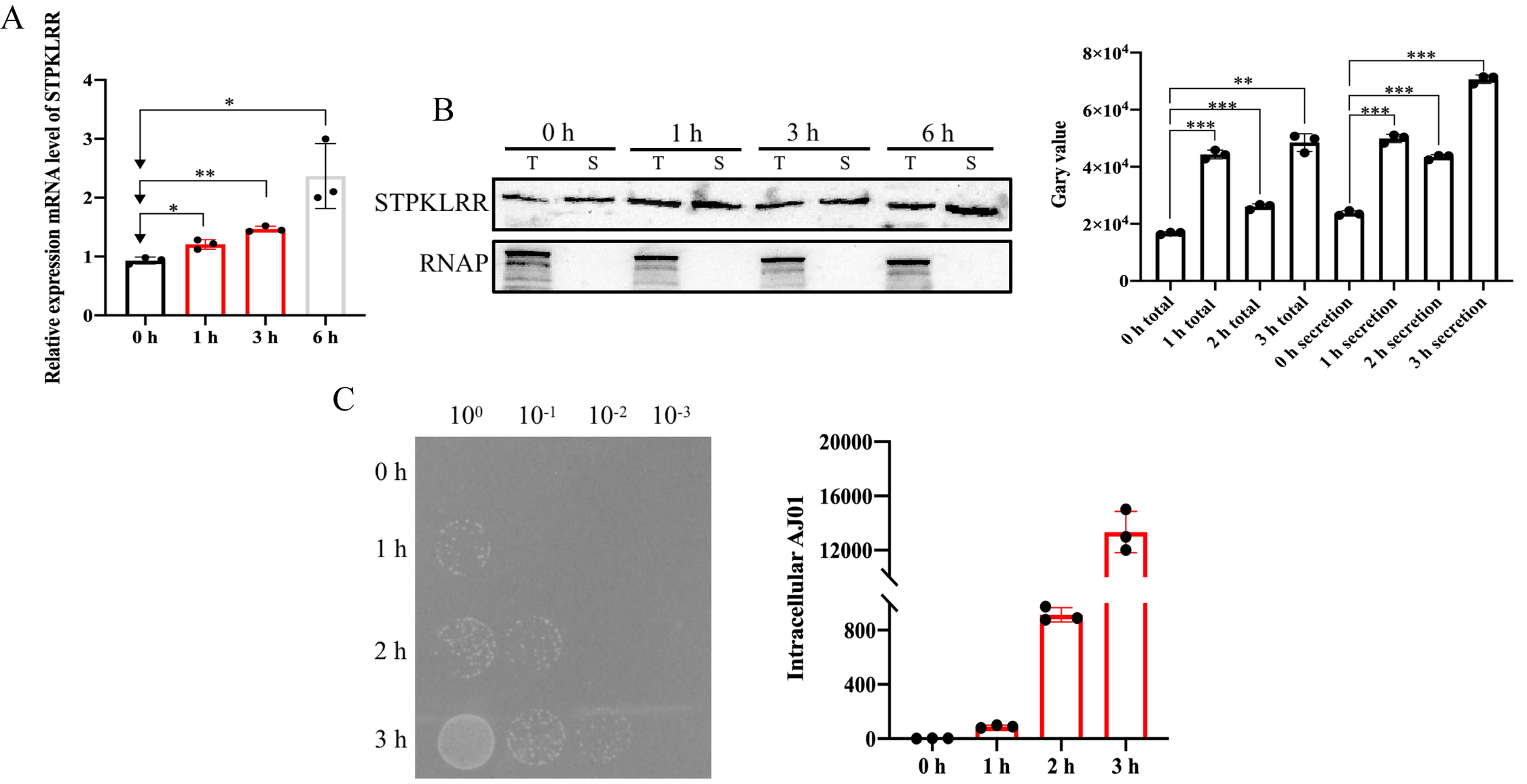

Supplement: S4 Fig — qRT-PCR (A) and western blotting analysis (B) of STPKLRR expression in sea cucumber coelom fluid treatment. RNAP, RNA Polymerase, was used as a bacterial cytosolic marker. The data, which are presented as the means ± SDs (n = 3) relative to the negative group, are shown in bar graphs, respectively. Asterisks indicate significant differences compared with the control group: ***p < 0.001 and **p < 0.01 (t-test). (C) Intracellular AJ01 burden was collected and plated on 2216E solid medium with gradient dilution (left panel). The single colonies on the 2216E solid medium were counted (right panel). (TIF) [file ppat.1011419.s004.tif]

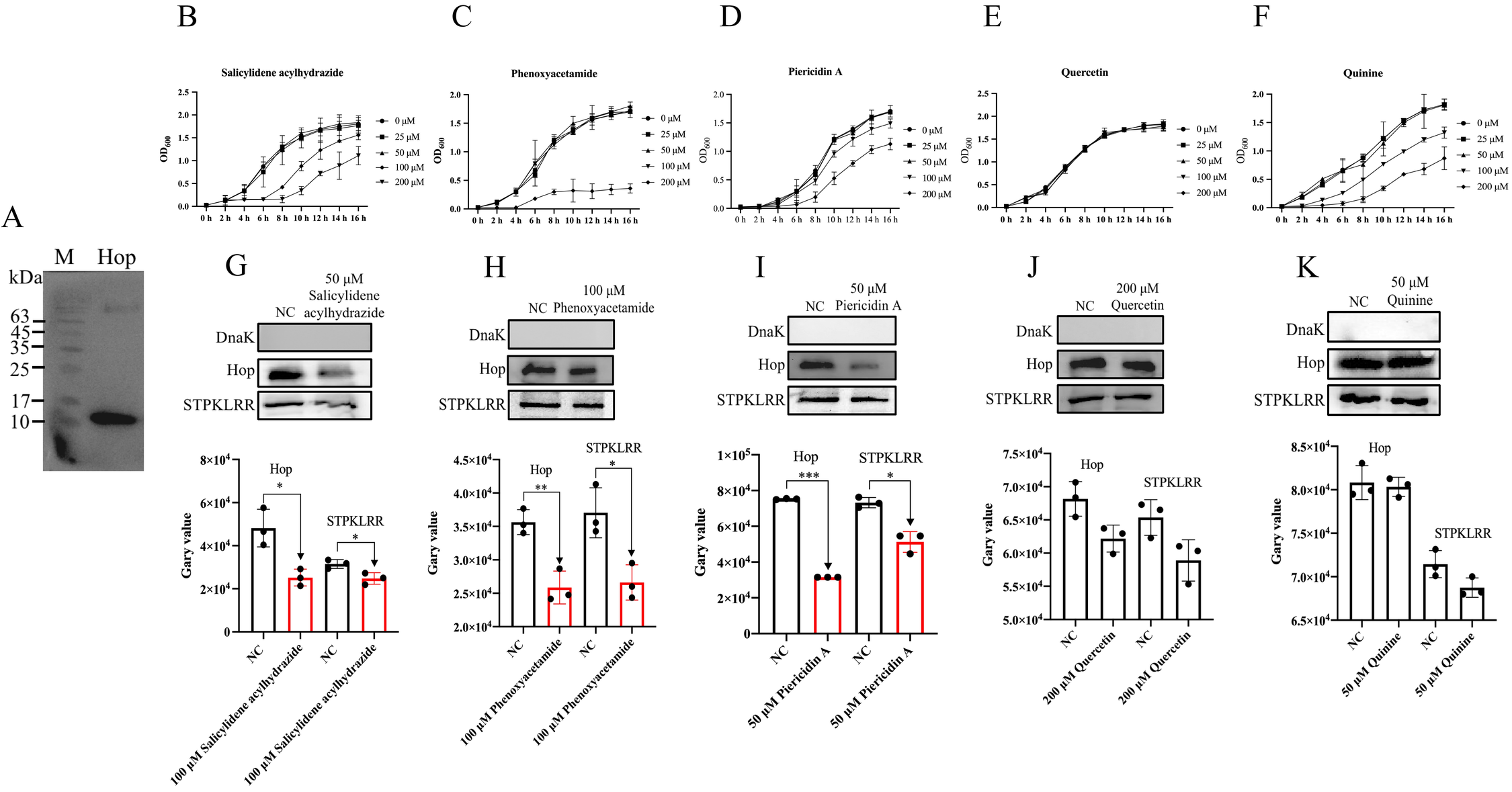

Supplement: S5 Fig — (A) Western blot analysis of the specificity of Hop antibody. The optimal concentration of Salicylidene acylhydrazide (B), Phenoxyacetamide (C), Piericidin A (D), Quercetin (E) and Quinine (F). The absorbance value of AJ01 with different concentrations of inhibitors was measured at 600 nm at each hour. The optimal inhibitor concentration that does not affect the growth of AJ01, was Salicylidene acylhydrazide 50 μM, Phenoxyacetamide 100 μM, Piericidin A 50 μM, Quercetin 200 μM, Quinine 50 μM, respectively. (G-K) After 16 h treatment of each inhibitor, the secreted proteins of AJ01 were extracted for western blotting. DnaK, a marker of bacterial cytosolic marker; Hop, a marker of vibrio T3SS secreted proteins. Band density was quantified using ImageJ, and protein levels of Hop and STPKLRR treated inhibitors were quantified and normalized to the NC group. Data (means ± SD) are representative of at least 3 experiments. Asterisks indicate significant differences (*p < 0.05; ***p < 0.001). (TIF) [file ppat.1011419.s005.tif]

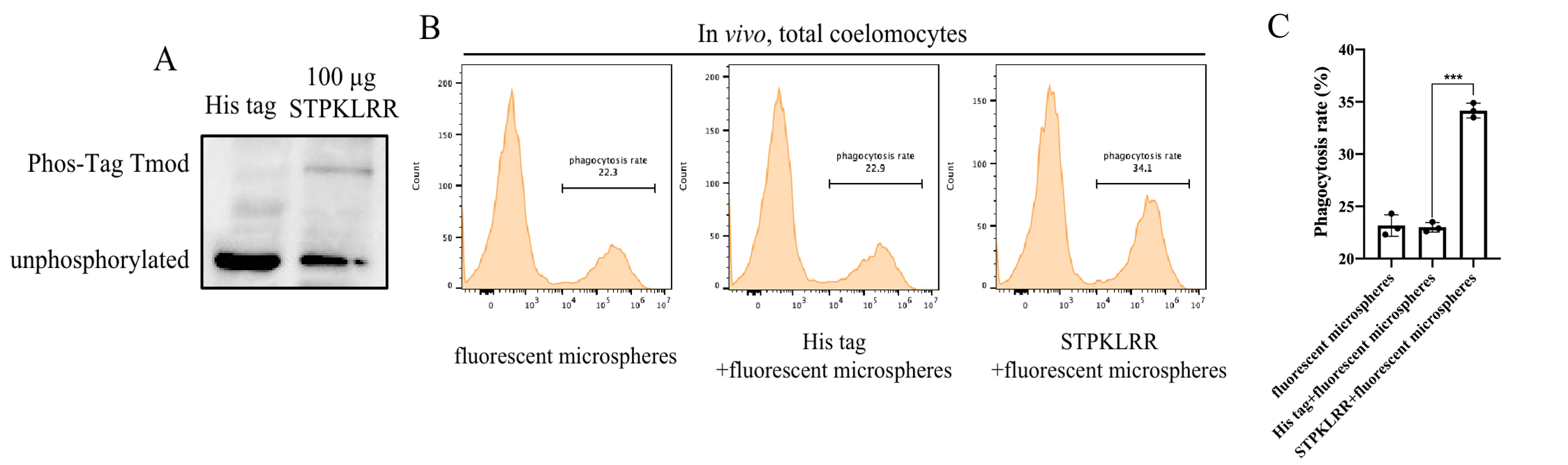

Supplement: S6 Fig — (A) Coelomocytes treated with 100 μg STPKLRR or His tag for 12 h were collected and used for the detection of AjTmod phosphorylation. (B) After treated with 100 μg STPKLRR or His tag for 12 h, 20 μL FITC-labeled fluorescent microspheres were added and incubated for another 3 h. (C) The coelomocytes phagocytosis rate of fluorescent microspheres was detected by flow cytometry. Data (means ± SD) are representative of at least 3 experiments. Asterisks indicate significant differences (***p < 0.001). (TIF) [file ppat.1011419.s006.tif]

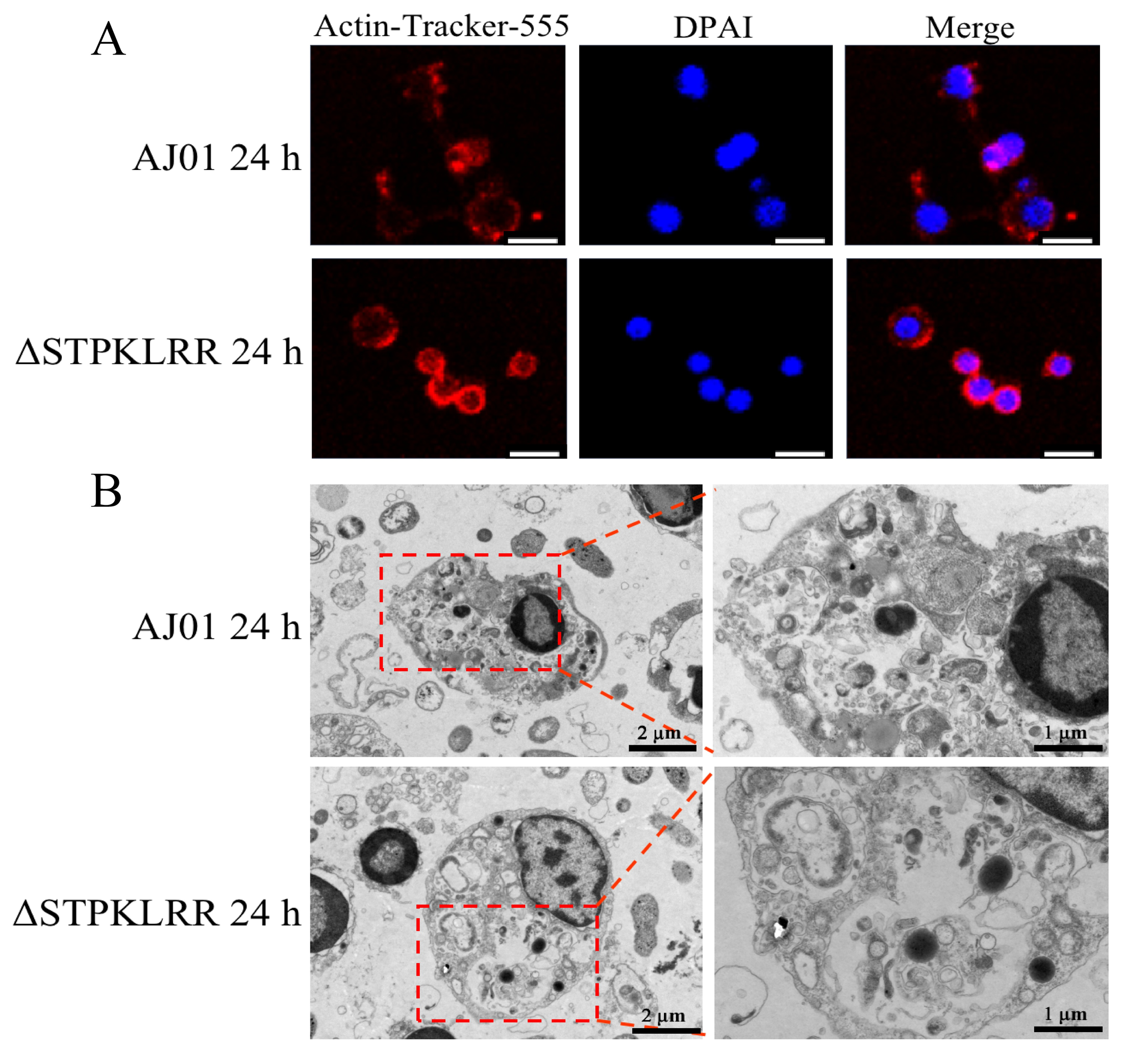

Supplement: S7 Fig — Coelomocytes were infected with AJ01 or ΔSTPKLRR (MOI = 100) for 24 h and collected for the immunofluorescence (A, Scale bar, 5 μm) and transmission electron microscope analysis (B, Scale bar, 2 μm, and 1 μm). (TIF) [file ppat.1011419.s007.tif]

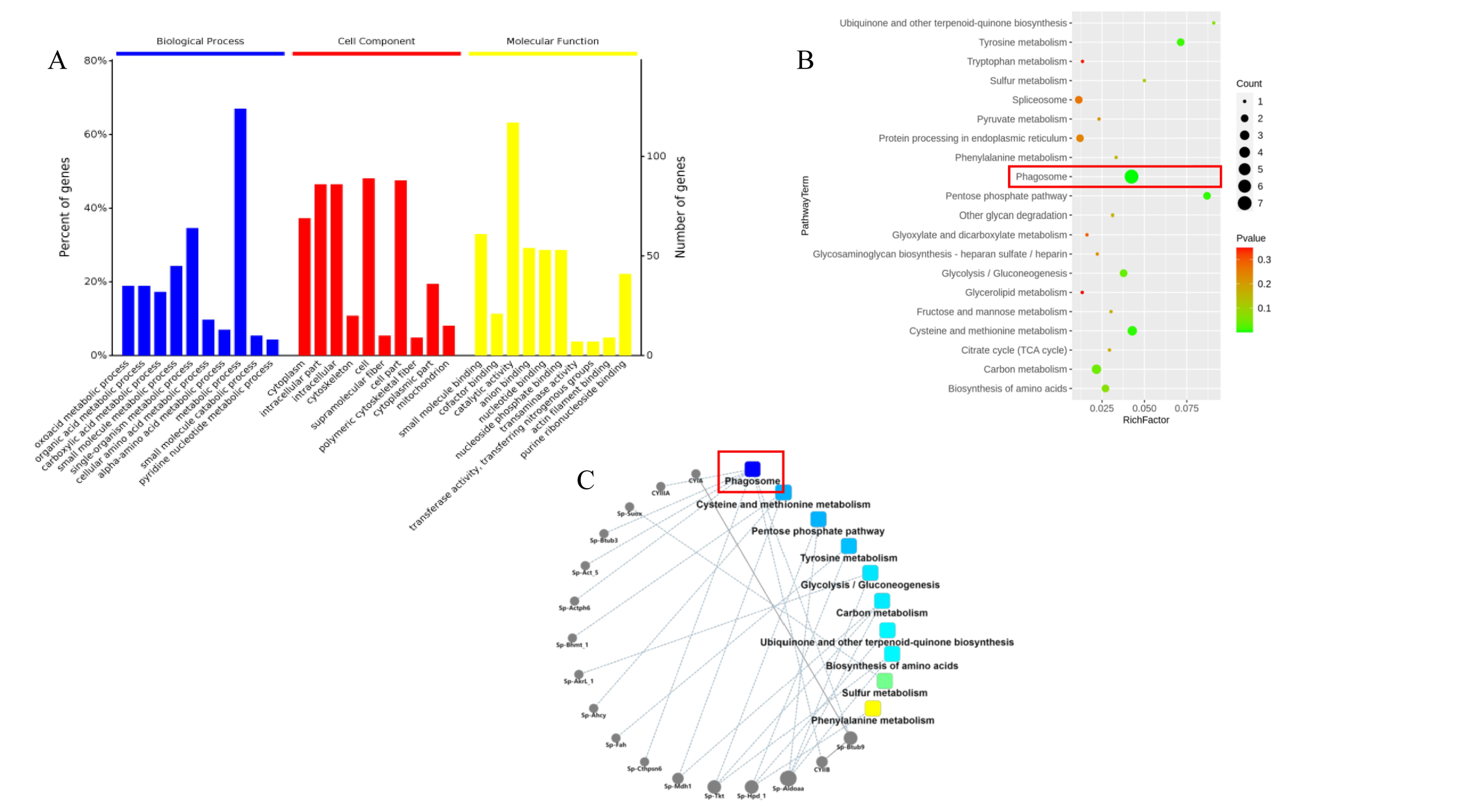

Supplement: S8 Fig — AjTmod-interacting proteins were identified by using a pull-down assay and further characterized by mass spectrometry to determine AjTmod-mediated processes in coelomocytes. The identified proteins were enriched in GO (A), KEGG (B), and PPI (C) analyses. (TIF) [file ppat.1011419.s008.tif]

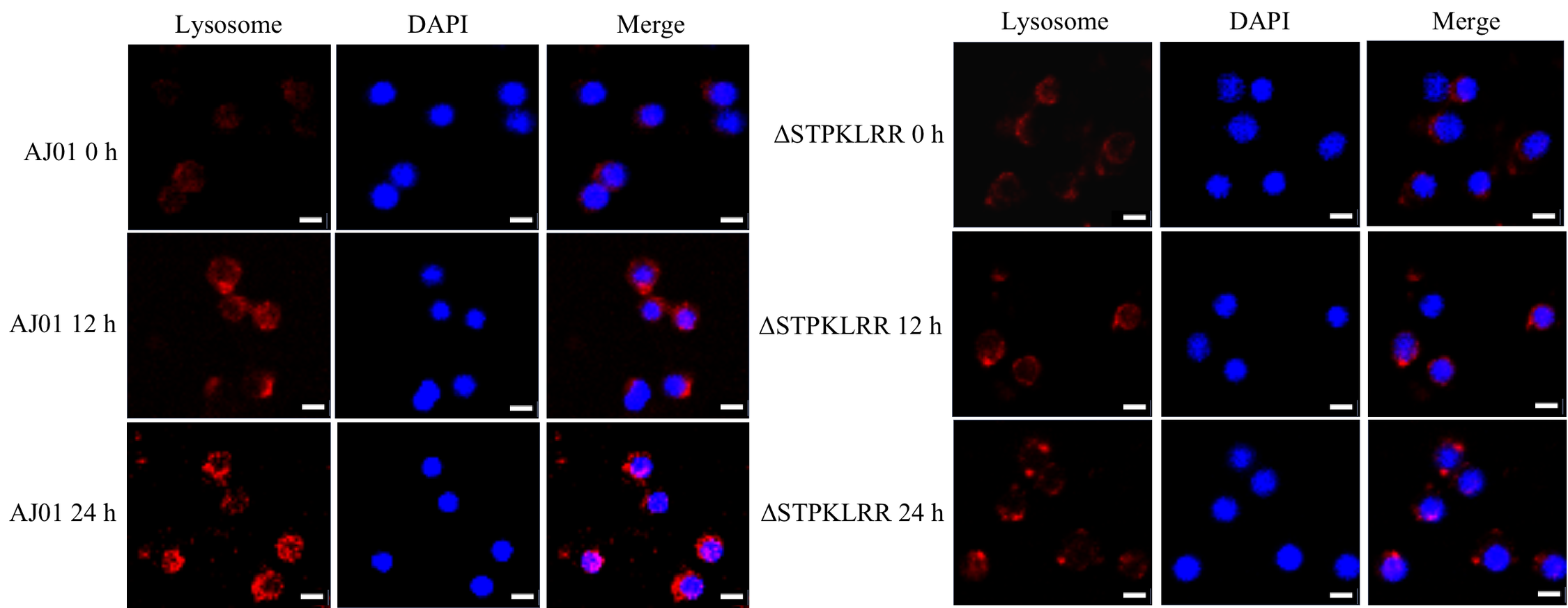

Supplement: S9 Fig — After AJ01 and ΔSTPKLRR 0 h, 12 h and 24 h infection, coelomocytes were collected, incubated with Lyso-Tracker Red to label the lysosomes, stained with DAPI and then observed under a laser-scanning confocal microscope. Scale bar, 5 μm. (TIF) [file ppat.1011419.s009.tif]
