# Supplementary material for: Novel secreted STPKLRR from Vibrio splendidus AJ01 promotes pathogen internalization via mediating tropomodulin phosphorylation dependent cytoskeleton rearrangement
Source: PLoS Pathog. 2023 May 22;19(5):e1011419. doi: 10.1371/journal.ppat.1011419 (PMC10237653; doi:10.1371/journal.ppat.1011419)
Supplement: Attachment — Submitted filename: Point-by-point response to reviewers.docx [file ppat.1011419.s015.docx]

Dear Editors,

We are submitting here a manuscript entitled “A novel secreted protein from *Vibrio splendidus* AJ01 promotes pathogen internalization via phosphorylating tropomodulin to mediate cytoskeleton rearrangement (PPATHOGENS-D-23-00489)”. We thank you and the reviewers’ constructive comments on our original submission, which were valuable and very helpful for revising and improving our paper and provided important guiding significance to our research. We have studied all the comments carefully and have made revision which marked in yellow color in the paper. All comments from the four reviewers have been addressed in our revise manuscript. All the required experiments have been added in our manuscript and supplementary materials. Below is a summary of the revisions and responses.

**Reviewer #1**: In this paper the authors identify a leucine-rich repeat-containing serine/threonine protein kinase from *Vibrio splendidus* AJ01. The authors use several methods to demonstrate that this protein is used by the pathogen to phosphorylate Tropomodulin (Tmod) in *Apostichopus japonicus*, leading to that Tmod dissociate from actin which result in rearrangement of the cytoskeleton enabling internalization of the pathogenic AJ01 bacterium. This is an important finding in order to understand the infection biology of *V. splendidus*. The findings are new and very interesting and is a continuation of previous studies by this group, and the authors have done a proper and thorough coverage of previous relevant studies in the literature.

**Major Issues:**

1. The data partly support the conclusions, but some more controls and/or explanations are needed, since some results are confusing. The authors have to explain their results in a clearer way and not use so many panels in each figure. Most of the panels in the figures are far too small and the text is nearly impossible to read, and therefore very difficult to evaluate. This is also true for the some of the supplementary figures. For example, figure S6A-C are not readable so either remove or magnify.

**Authors response:** We thank the reviewer for these comments and add some necessary control in our revised manuscript. We apologized for any inconvenience caused by the previous oversight regarding the images. We had taken corrective measures by adjusting all images. To reduce the number of panels, minor figures had been placed in accompanying figures, while the remaining figures had been enlarged for easier reading. Original Figure S6A-C had been moved to Figure S8.

2. For figure 1D an explanation is needed in the legend about the details of the upper and lower panels. The figure should indicate His-STPKLRR and not only SPKLRR. Moreover, the pattern of the lanes in the blot for His-staining differ a lot from the blots for GST staining. Is the same amount of protein loaded on these gels? Please indicate the loading amount. The panels in figure 1 is also very small making them difficult to read and evaluate. I suggest that the authors place figure 1A-C as a supplement, and enlarge figure 1D.

**Authors response:** We thank the reviewer for these comments and improve Figure 1 based on your comments. We re-described the legend of Figure 1D in detail (Line 965-982) and corrected STPKLRR to HisSTPKLRR in Figure 1D. In the pull-down experiment, we used the same amount of loading protein (10 μg for each gel). Due to the difference in protein size for GST-staining and His-staining panel, the exposure time and image adjustment were different each group, which may be the reason for the patterns difference between the two panels. We rescaled Figure 1 to make each figure clearly visible in our revised manuscript.

3. Figure 2D is to low resolution to be understandable. It is not possible to understand what the arrow points at. There are too many panels in this figure as well. In figure 2G the number of intracellular AJ01 is shown, but to what are these figures related? Is it per gram tissue or something else?

**Authors response:** We appreciate the reviewer’s comments and provide Figure 2C with higher resolution replaced the original Figure 2D. We magnified the significantly diseased tissue and moved the redundant images to Figure S3B. The black arrow represented the tissue lesion area, which was pointed out in the legend. In the revised Figure 2, the statistics analysis of intracellular bacteria number in Figure 2E was showed in Figure 2F. The number of intracellular bacteria represented the number of intracellular bacteria in each well of a 6-well plate, and the number of cells per well was about 10^6^.

4. Another question is to the following experiments where coelomocytes are used. Are these cells the main target of this bacterium? In figure 2E second row the bacterium is in a phagosome, but is it killed there or does it multiply in coelomocytes?

**Authors response:** We appreciate the reviewer’s valuable comments. Previous studies had shown that coelomocytes were very important immune organs of *A. japonicus* (Lv et al., J Immunol., 2022;208: 464-479). Our previous experiments showed that coelomic fluids were most colonized by AJ01 (Dai et al., Aquaculture, 2020;523: 735169), suggesting that coelomocytes were indeed the main target of AJ01. It was hard to judge the fate of the AJ01 inside the phagosome. Generally speaking, a large part of intracellular bacteria would be degraded by phagosomes and lysosomes, but some bacteria could escape from phagosomes, which would be investigated in our further work.

5. Figure 3 has too many panels, which are too small to be readable. Magnification does make the panels blurry. The text in figure 3E-H is impossible to read. Figure 3A-B shows secretion of STPKLRR and uptake (?) into coelomoctes. The legend is a bit unclear. In 3A marker lanes for the western blot is needed, and for 3B the legend should indicate that the pictures show coelomocytes and internalization of STPKLRR (if this is correct).

**Authors response:** We appreciate the reviewer’s comments. We condensed Figure 3 and moved some images to Figure S5. We are sorry for the misunderstanding in the description of Figure 3. In Figure 3A, the total and secreted proteins were isolated from AJ01 rather than coelomocytes, which was used for STPKLRR localization analysis. DnaK was used as a marker of bacterial intracellular proteins. Figure 3B showed the internalization of secreted STPKLRR into coelomocytes. Translocation of STPKLRR into the coelomocytes resulted in the cleavage of CCF4-AM, causing the emission of blue fluorescence. Uncleaved CCF4-AM emitted green fluorescence. We were sorry for the incomplete description of the legend, and re-described Figure 3A and 3B (Line 997-1005).

6. Figure 3D shows coelomocyte extract from cell infected with the different mutants and “wild type” AJ01 strains used in figure 2, and STPKLRR is only found in the wild type infected cells or the “restored” ΔSTPKLRR::pMPSTPKLRR infected cells. However, why is DnaK detected in the cytoplasm of all coelomocytes if was necessary for bacterial internalization as shown in figure 2?

**Authors response:** We thank the reviewer for this comment. DnaK, as a marker of bacterial intracellular proteins, was used as a control for AJ01 internalization, which was not involved in AJ01 internalization. The loss of STPKLRR lead to the reduction of AJ01 internalization, but it would not lead to the complete disappearance of intracellular AJ01, so the DnaK signal could still be detected in the coelomocytes.

7. Figure 3E-G: Explain that these figures show the effect of the T3SS inhibitors on bacterial growth (if this is correct). These panels could be moved to supplemental material.

**Authors response:** We agree with the reviewer’s comments and move these figures to Figure S5.

8. The DnaK panels in figure H-J looks completely empty (non loaded), and moreover the non-existent variability of the band intensity shown in the diagrams 3H-J looks very strange. Is it really possible to get nearly identical blots from three different biological replications? What does Gary value mean?

**Authors response:** We appreciate the reviewer’s comments. DnaK was a bacterial intracellular protein and not present in secreted proteins. Therefore, DnaK could not be detected in secreted proteins in Figure 3H-3J, similar to Figure 3A. We provided three biological replicates results for STPKLRR and DnaK (Figure 1, below). The Gary value represented the blot intensity. ImageJ software was used to analyze the blot intensity with the wand tool.

**Figure 1.** Triplicate expression of STPKLRR and Hop protein levels under different inhibitors treatment. **(A, B)** After 16 h treatment of each inhibitor, the secreted proteins of AJ01 were extracted for Western blot. #1, #2 and #3 are three repeats of STPKLRR and Hop respectively. **(C-E)** Band density was quantified using ImageJ, and protein levels of Hop and STPKLRR treated inhibitors were quantified and normalized to the NC group. Data (means ± SD) are representative of at least 3 experiments. Asterisks indicate significant differences (**p* < 0.05; ***p* < 0.01).

9. I am a bit concerned over figure 4A (and 4B), since the phosphorylated bands look pasted, and have moved in a straight manner compared to the non-phosphorylated proteins. The other panels in figure 4, except for E, F and L are too small to evaluate. The concentrations used in figure 4K are not visible, and are important to judge the results.

**Authors response:** We appreciate the reviewer’s comments. To avoid the misunderstanding, we repeated the experiment with increased protein loading, and the revised Figure 4A and 4B were provided in our revised manuscript. In the revised Figure 4A, the loading proteins were increased from the original 20 μg to 50 μg and the results also indicated that STPKLRR mediated one site phosphorylation of AjTmod. In revised Figure 4B, we re-performed the *in vitro* protein kinase activities assay and the Figure of Phos-Tag SDS-PAGE was revised to detect phosphorylated and non-phosphorylated Tmod at the same time. We also divided the original Figure 4 into the two figures to make the figures more readable. The binding reaction system of MST was marked in our revised Figure 5G.

10. In figure 5H the total amount of actin varies between time points. Is this correct?

**Authors response:** We appreciate the reviewer’s comments. The F-actin/G-actin ratio was determined in accordance with a previously reported method (Huang et al., Nat Neurosci., 2013;16: 441-8). Following the steps, we extracted protein samples of F-actin and G-actin at various time points, and then quantified with BCA. The total sample loading of 30 μg was used for western blot analysis. This problem of F/G-actin ratio in WB was a common phenomenon (Reddy et al., PLoS One, 2013;8:e73763; Kim et al., Am J Physiol Cell Physiol., 2008;295:C768-78; Cook et al., PLoS One, 2014;9:e89847), which might be caused by inaccurate protein quantification of some samples or other factors.

**Minor Issues:**

1. The authors need to improve the quality of all the small panels and arrange the paper in a readable way. The English also needs professional editing.

**Authors response:** We thank the reviewer for these comments and correct it in our revised manuscript. Also, the manuscript has been edited by ShineWrite.com professional editing support with ID of 40-2022121114243374 (http://www.shinewrite.com/). Special thanks for the reviewer’s comments.

**Reviewer #2:** This report is an important contribution to our understanding about the detailed mechanisms used by *Vibrio splendidus* to enter *Apostichopus japonicus* coelomocytes. The authors provide clear evidence for the role of the serine/threonine kinase activity of STPKLRR, to phosphorylate tropomodulin in the coelomocyte cytoplasm, in order to favor *V. splendidus* internalization by phagocytosis most likely through macropinocytosis and actin-dependent endocytic pathways.

Tropomodulin in *Apostichopus japonicus* was first characterized in the group’s previous work in JBC, which plays an important role in coelomocytes apoptosis. Based on the first publication, the authors identify a Tmod-targeted mechanism of cell entry in *Vibrio splendidus* with a considerable number of assays. This makes sense from a pathogen-host interaction perspective.

The role of Tmod in stabilizing the cytoskeleton seems to be its own job. However, Tmod plays a role in apoptosis, which is not discussed in the manuscript. The multifunctionality of a single gene is not surprising, but the involvement of pathogen-host interaction at the same time is worthy of attention in the follow-up research of the authors.

**Major Issues:**

1. The morphology of intracellular bacteria in the second and fifth panels of Figure 2E is obviously different. Why is the case? Is this due to the introduction of the vector or other bacteria? If not, some plausible explanation is needed to demonstrate the possible cause of the intracellular morphological changes in Vibrio splendidus.

**Authors response:** We thank the reviewer for this comment. Morphological plasticity of bacteria was a cryptic phenomenon, by which bacteria acquired adaptive benefits for coping with changing environments. Physical and chemical factors causing morphological changes in bacteria had been investigated and mostly associated with potential pathways linked to the cell wall synthetic machinery (Shen et al., Biomicrofluidics, 2016;10: 031501). These include starvation, oxidative stresses, predation effectors, antimicrobial agents, temperature stresses, osmotic shock, and mechanical constraints. Thus, morphological changes in bacteria were a common phenomenon (Justice et al., Nat Rev Microbiol., 2008;6: 162-8). Tran et al. found that after adding the inhibitor IWP-2 of bacterial transcriptional activator PrfA, the morphology of the bacteria showed a significant difference (Tran et al., PLOS Pathog., 2022;18: e1010166). Infection time differences could result in changes to bacterial morphology. Research had shown that *Pseudomonas aeruginosa* exhibited differences in morphology after 30 minutes and 3 hours of infection (Garai et al., PLOS Pathog., 2019;15: e1007812). In our experiment, isolated intracellular bacteria were characterized by 16S rDNA sequencing, which was identical to *Vibrio splendidus*. The morphology in Figure S3C (Original Figure 2E) differed from that of the typical intracellular AJ01, which portrayed AJ01 replicating in the coelomocyte.

**Minor Issues:**

1. Line 61: The provided references of MARTX in *Vibrio cholerae* are wrong. Please provide the correct references.

**Authors response:** We are sorry for our mistake and provide the correct reference.

2. - Line 77: "Tmod, the only known capping protein of F-actin" is inaccurate. It is the only pointed (minus) end-capping protein.

**Authors response:** We appreciate the reviewer’s comments and revised the sentence "Tmod, the only known capping protein of F-actin" to "Tmod, the only pointed end-capping protein of F-actin" (Line 86).

3. Check spellings: β-Tubulin or β-tubulin? It should be unified. In Fig. 5, coleomocytes should be corrected to coelomocytes.

**Authors response:** We are sorry for our mistake and have thoroughly examined the entire manuscript and addressed these issues.

4. In Fig. 2 and Fig. 3, there are some panels with small text. They are difficult for a reader to judge.

**Authors response:** We appreciate the reviewer’s comments. After incorporating the suggestions of all reviewers, we have made adjustments to all the figures to ensure legibility.

5. Figure S6 can better connect the logic of the manuscript. It is suggested to move it to the main text of the manuscript.

**Authors response:** We agree with the reviewer’s comments. Original Figure S6 has been converted to Figure 6.

**Reviewer #3:** This manuscript mainly revealed the mechanisms that a novel *Vibrio splendidus* AJ01 Type III secretion system effector of STPKLRR could specifically interact with AjTmod, causing the phosphorylation of AjTmod at serine 52, thus reducing the binding stability between AjTmod and actin. After AjTmod dissociated from actin, the F-actin/G-actin ratio decreased to induce cytoskeletal rearrangement, which in turn promoted the internalization of AJ01. Supported by a large number of experimental data, this manuscript answers how AJ01 breaks the AjTmod-stabilized cytoskeleton for internalization from fine details.

**Major Issues:**

1. Is STPKLRR located on the surface of AJ01? How does AJ01 expose STPKLRR to bind Tmod during internalization?

**Authors response:** We appreciate the reviewer’s comments. As shown in Figure 3, STPKLRR was secreted by AJ01 type III secretion system (T3SS). It was not located on the bacterial membrane, but it was injected into the coelomocytes through T3SS in the early stage of infection, and after entering the coelomocytes, STPKLRR targeted and phosphorylated Tmod.

2. Does STPKLRR-mediated AJ01 internalization promotes the phagocytosis of AJ01? Phagocytosis is a strategy for eliminating pathogens, but STPKLRR-mediated AJ01 internalization promotes AJ01 infection, which seems to contradict each other in this manuscript. It is recommended to test lysosomal activity.

**Authors response:** We agree with the reviewer’s comments. Lysosome activity was detected with Lyso-Tracker Red (Beyotime, China) at 12 h and 24 h after AJ01 and ΔSTPKLRR infection. The lysosome activity of ΔSTPKLRR was found to be significantly lower than that of the AJ01 group, indicating that STPKLRR-mediated internalization of AJ01 promoted the activation of coelomocyte phagocytosis-lysosome (Figure 2, below).

Activation of phagolysosomes was an effective method for eliminating intracellular pathogens, despite the fact that it appeared to contradict bacterial internalization. Currently, intracellular bacteria such as *Mycobacterium tuberculosis*, *Listeria monocytogenes*, *Shigella*, and *Salmonella* had been found to utilize various methods to escape intracellularly, including inhibiting phagosome acidification, destroying phagosome membranes, and inhibiting phagosome-lysosome binding (Bianchi et al., J Cell Sci., 2020;134: jcs247221). For AJ01, internalization into coelomocytes was probably just the beginning in the host-bacteria interaction, and immune escape in coelomocytes was the key to its successful infection. Our follow-up work would also focus on the immune escape of AJ01.

**Figure 2.** The lysosome activity of coelomocytes infected with AJ01 and ΔSTPKLRR. After AJ01 and ΔSTPKLRR 0 h, 12 h and 24 h infection, coelomocytes were collected, incubated with Lyso-Tracker Red to label the lysosomes, stained with DAPI and then observed under a laser-scanning confocal microscope. Scale bar, 5 μm.

**Minor Issues:**

1. Fig. 3C, the luminance of DAPI is inconsistent. Is the setting of fluorescence parameters inconsistent? In addition, STPKLRR seems to affect the expression of Tmod, but this experiment is not mentioned in this paper.

**Authors response:** We appreciate the reviewer’s comments and have corrected the DAPI fluorescence intensity in Figure 3C. In our previous work, the increase of AjTmod transcription and protein level expression was caused by the infection of AJ01 (Dai et al., J Biol Chem., 2022;298: 102091). We re-conducted the experiment to see whether STPKLRR affects the expression of AjTmod. As shown in below Figure 3, the expression of Tmod significantly increased after 12 h and 24 h AJ01 and ΔSTPKLRR infection. However, there were no significant difference between the two groups at each time point, suggesting that STPKLRR did not mediate the expression of Tmod.

**Figure 3.** The protein expression of AjTmod. **(A)** Western blotting analysis of Tmod expression at 0 h, 3 h, 12 h, and 24 h AJ01 or ΔSTPKLRR infection. **(B)** Band density was quantified using ImageJ. Data (means ± SD) are representative of at least 3 experiments. Asterisks indicate significant differences (* *p* < 0.05; ** *p* < 0.01).

2. Fig. 4A, lacking of internal reference, so there is no comparability between different experimental groups.

**Authors response:** We appreciate the reviewer’s comments. Incorporating the first reviewer’s comments, we revised Figure 4A and added internal references.

3. Fig. 4F, it is recommended to add STPKLRR signal.

**Authors response:** We thank the reviewer for these comments. Figure 3A showed that the signal of STPKLRR co-localizes with Tmod in the early stage of infection. In Figure 5A, Tmod, actin and STPKLRR were also successfully co-localized. Combined with other results, it could be shown that STPKLRR mediated the dissociation of the Tmod/actin complex. Continuing to track the intracellular localization of STPKLRR could not better justify and enrich the current conclusions. As a secreted effector, STPKLRR was mainly injected into coelomocytes by AJ01 to regulate the cytoskeleton in the early stage of infection. Whether STPKLRR would perform new functions after AJ01 internalized into coelomocytes should be investigated in our follow-up work.

4. Fig. 5, although F-actin/G-actin ratio indicates cytoskeleton rearrangement, it is suggested to add immunofluorescence assay to observe cytoskeleton changes.

**Authors response:** We thank the reviewer for these comments. In Figure S7, we have observed STPKLRR-mediated F/G-actin-dependent cytoskeletal rearrangements by immunofluorescence assay and transmission electron microscopy, respectively.

**Reviewer #4:** This is an interesting study which adds to advancing our understanding of TSS3 mechanisms of *Vibrio splendidus* in the host *Apostichopus japonicus*. The study is comprehensive, with a full range of different advanced methods being employed to provide insights of mechanisms. The general execution and scholarship appear sound. However, the resolution and size (and complexity) of the multi panel figures completely defeats review. Image resolution is so poor - it is impossible to judge the detail of what is being shown, or follow the interpretation or verify claims.

I would advise images are submitted at higher resolution, and would actually encourage the submission of simpler figures with fewer panels per composite figure.

**Major Issues:**

I would not recommend that further experiments are necessary.

**Minor Issues:**

Resolution of figures must be dramatically improved. For example, the histopathology shown in figure 2 is impossible to judge. E.g. figure 4 is too small, and too low resolution to follow. Etc.

**Authors response:** We thank the reviewer for these comments. Incorporating comments from all reviewers, we minimized the figure panels and enlarged figures to make the figures clarity and readability. Special thanks for the reviewer’s comments.

We have done our best to improve our manuscript and made changes wherever necessary. These changes do not influence the content and framework of our paper. We thank the Editors/Reviewers for their time and hope that the revised manuscript can now be approved for publication.

Looking forward to your favorable decision.

Best regards

Chenghua Li

**Related Reference**

Bianchi F, van den Bogaart G. (2020) Vacuolar escape of foodborne bacterial pathogens. J Cell Sci.134(5): jcs247221. doi: 10.1242/jcs.247221. PMID: 32873733

Dai F, Guo M, Shao Y, Li C. (2022) *Vibrio splendidus* flagellin C binds tropomodulin to induce p38 MAPK-mediated p53-dependent coelomocyte apoptosis in Echinodermata. J Biol Chem. 298: 102091. doi: 10.1016/j.jbc.2022.102091. PMID: 35654141

Garai P, Berry L, Moussouni M, Bleves S, Blanc-Potard AB. (2019) Killing from the inside: Intracellular role of T3SS in the fate of *Pseudomonas aeruginosa* within macrophages revealed by mgtC and oprF mutants. PLoS Pathog. 15(6): e1007812. doi: 10.1371/journal.ppat.1007812. PMID: 31220187

Justice SS, Hunstad DA, Cegelski L, Hultgren SJ. (2008) Morphological plasticity as a bacterial survival strategy. Nat Rev Microbiol. 6(2): 162-8. doi: 10.1038/nrmicro1820. PMID: 18157153

Lv Z, Guo M, Zhao X, Shao Y, Zhang W, Li C. (2022) IL-17/IL-17 receptor pathway-mediated inflammatory response in *Apostichopus japonicus* supports the conserved functions of cytokines in invertebrates. J Immunol. 208(2): 464-479. doi: 10.4049/jimmunol.2100047. PMID: 34965964.

Shen JP, Chou CF. (2016) Morphological plasticity of bacteria-Open questions. Biomicrofluidics. 10(3): 031501. doi: 10.1063/1.4953660. PMID: 27375812

Tran TT, Mathmann CD, Gatica-Andrades M, Rollo RF, Oelker M, Ljungberg JK, et al. (2008) Inhibition of the master regulator of Listeria monocytogenes virulence enables bacterial clearance from spacious replication vacuoles in infected macrophages. PLoS Pathog. 18(1): e1010166. doi: 10.1371/journal.ppat.1010166. PMID: 35007292
